# Potentiation of Insulin-Mediated Glucose Lowering without Elevated Hypoglycemia Risk by a Small Molecule Insulin Receptor Modulator

**DOI:** 10.1371/journal.pone.0122012

**Published:** 2015-03-23

**Authors:** Margaret Wu, Ge Dai, Jun Yao, Scott Hoyt, Liangsu Wang, James Mu

**Affiliations:** Early Development and Discovery Sciences, Merck Research Laboratories, Merck Sharp & Dohme Corp., Whitehouse Station, NJ 08889, United States of America; Institut d'Investigacions Biomèdiques August Pi i Sunyer, SPAIN

## Abstract

Insulin resistance is the key feature of type 2 diabetes and is manifested as attenuated insulin receptor (IR) signaling in response to same levels of insulin binding. Several small molecule IR activators have been identified and reported to exhibit insulin sensitization properties. One of these molecules, TLK19781 (Cmpd1), was investigated to examine its IR sensitizing action *in vivo*. Our data demonstrate that Cmpd1, at doses that produced minimal efficacy in the absence of insulin, potentiated insulin action during an OGTT in non-diabetic mice and enhanced insulin-mediated glucose lowering in diabetic mice. Interestingly, different from insulin alone, Cmpd1 combined with insulin showed enhanced efficacy and duration of action without increased hypoglycemia. To explore the mechanism underlying the apparent glucose dependent efficacy, tissue insulin signaling was compared in healthy and diabetic mice. Cmpd1 enhanced insulin’s effects on IR phosphorylation in both healthy and diabetic mice. In contrast, the compound potentiated insulin’s effects on Akt phosphorylation in diabetic but not in non-diabetic mice. These differential effects on signaling corresponding to glucose levels could be part of the mechanism for reduced hypoglycemia risk. The *in vivo* efficacy of Cmpd1 is specific and dependent on IR expression. Results from these studies support the idea of targeting IR for insulin sensitization, which carries low hypoglycemia risk by standalone treatment and could improve the effectiveness of insulin therapies.

## Introduction

As the insulin resistant and diabetic population expands worldwide at an increasing pace, insulin analogs and insulin sensitizers continue to be highly demanded therapeutic options. Collectively, they represent some of the most potent glucose lowering agents and a large portion of worldwide anti-hyperglycemic agent prescriptions [1]. Since the introduction of purified insulin to clinical use nearly a century ago, there have been continuous efforts to develop analogs [2] mimicking physiological insulin action, including rapid acting and basal insulin analogs. However, concerns around hypoglycemia and other risks associated with self-injection often limit optimal insulin efficacy and prevent patients from achieving ideal glycemic control [3]. As a consequence, safer and more convenient administration of insulin and effective insulin sensitizers are more desirable, especially as the global population is becoming more insulin resistant and typical insulin dosages are shifting higher. Despite that need, identifying bona fide and clinically efficacious insulin sensitizer targets carrying no liabilities has been challenging. One recent example is the PPARγ activator, which increases insulin sensitivity and lowers glucose effectively, but is associated with significant body weight gain and cardiovascular risk [4].

Insulin mimetics, including novel peptides bearing no sequence homology with insulin [5–7], monoclonal antibodies [8], and small molecule [9–18] IR activators, have been explored as alternative approaches to activate IR. Among them, several small molecule IR activators have been shown to exhibit glucose lowering potency *in vivo* [10,13,15,18,20]. While a non-parenteral IR activator could help to improve ease of administration and patient compliance compared with injectable insulins, the concern over hypoglycemic risk remains, and may limit its potential benefit. Interestingly, some studies have shown that selected small molecule IR activators can bind to IR non-competitively with insulin and demonstrate potential insulin sensitizing action [10,13,20]. Unlike orthosteric IR activators, ligands that bind allosterically to IR and sensitize insulin action would represent a significant therapeutic advancement with the potential of alleviating insulin resistance and minimizing hypoglycemia risk. For example, type 2 diabetic patients could use their own insulin more effectively, while type 1 patients may be able to achieve comparable glucose lowering with lower doses of insulin. This could translate to fewer instances of adverse effects, such as hypoglycemia and body weight gain, increased patient compliance, and ultimately improved glycemic control.

In a search for IR activators, a set of small molecules was identified by affinity screening using the IR intracellular domain [10,12,21]. Representative molecules from the series were shown to activate insulin signaling and enhance insulin action in cell-based assays. When dosed alone, they lowered glucose in diabetic mice [10,13,20]. To explore the feasibility of identifying allosteric IR small molecule modulators as insulin sensitizers, we studied TLK19781 ([22]; Cmpd1) in combination with insulin in several mouse models. Our results show Cmpd1 does not cause significant glucose lowering by itself, but can robustly sensitize insulin action when glucose levels are high. As expected from a true insulin sensitizer, Cmpd1 does not increase hypoglycemia as an IR activator would. Interestingly, the Cmpd1-mediated increase in insulin signaling appears to be glucose dependent at the level of Akt phosphorylation, which could explain the improved efficacy vs. safety profile of the insulin and Cmpd1 combination.

## Materials and Methods

### Ethics Statement

All animal procedures were reviewed and approved by the Institutional Animal Care and Use Committee of Merck Research Laboratories, NJ, USA. The Guide for the Care and Use of Laboratory Animals was followed in the conduct of the animal studies. Veterinary care was given to any animals requiring medical attention.

### Materials

All chemicals and reagents were procured from commercial sources except for Cmpd1 [22], which was synthesized in-house.

### Animals

Male C57BL/6 mice (8 weeks of age) were obtained from Taconic Farm (Germantown, NY, USA) and housed eight per cage in a room maintained at constant temperature (25°C). Male db/db mice were purchased at 5 weeks of age from Jackson laboratory (Bar Harbor, ME, USA) and housed eight per cage in temperature, humidity, and light controlled rooms with *ad libitum* access to autoclaved water and food (Diet 5008, Purina, Framingham, MA, USA). To generate streptozotocin (STZ)-induced diabetic mice, adult C57BL/6 male mice were treated with a single intraperitoneal (i.p.) injection of either vehicle (saline) or STZ (Sigma Chemical, St. Louis, MO, USA) at 100–125 mg/kg [23]. At the beginning of the experiment, db/db (8 weeks of age) or STZ-diabetic mice (10 weeks of age) were grouped based on body weight and ambient glucose levels.

A mouse model of reversible insulin receptor knockdown with expression of an IR-specific shRNA was obtained from Taconic Farm (Germantown, NY, USA). Hyperglycemia and hyperinsulinemia were induced by feeding adult mice with doxycycline (20 ug/ml; sigma D-9891, Sigma Chemical, St. Louis, MO, USA) in water sweetened with 1% Sucrose [24].

Unless otherwise specified, mice were maintained under controlled conditions of lighting (12 h light/dark), temperature (23 ± 2°C), and humidity (55 ±15%) with access *ad libitum* to rodent diet (7012 Teklad LM-485; Harlan Laboratories, Indianapolis, IN, USA) and water.

### Glucose Tolerance Test

C57BL/6 mice at 8 weeks of age were fasted for 4 hours and injected i.p. with 10 mL/kg cmpd1(30 mg/kg) 30 minutes prior to an oral glucose dose at 5 g/kg of body weight and an i.p. injection of 0.5 U/kg insulin. Glucose levels were measured from tail bleeds with a OneTouch glucometer (Lifescan, Milpitas, CA; USA) at specified time points after glucose administration [25]. In a separate cohort of mice studied in parallel (to avoid impact of bleeding-induced stress on glucose measurements), plasma samples were collected 0, 30 and 60 minutes post insulin dosing. C-peptide (ELISA, Alpco, Windham, NH, USA) and glucagon levels (Meso Scale Discovery, MD, USA) were measured according to manufacturer’s protocols.

### Evaluations of Antihyperglycemic Efficacy in C57BL/6, *db/db* and STZ-diabetic Mice

Acute glucose lowering was studied in 2 hour fasted mice administered a single i.p. injection of 30 mg/kg cmpd1 in 10 mL/kg PBS and i.p. injection of various insulin doses (0.3–1 U/kg to STZ mice; 0.6–2 U/kg to db/db mice; 1–3 U/kg to normoglycemic C57BL/6 mice to evaluate hypoglycemia risk). Animals were fasted during the interval between dosing and the final blood glucose measurement. Glucose was measured in blood from tail bleeds using a OneTouch glucometer at specified time points.

### Phospho-IR and Phospho-Akt Measurement

Healthy normoglycemic C57BL/6 mice and age-matched STZ-diabetic mice were studied under the same conditions to compare insulin signaling using phospho-IR (pIR) and phospho-Akt (pAkt). Cmpd1 (30 mg/kg) was dosed i.p. 4 hours after food removal together with Humulin R (1 U/kg, Eli Lilly, IN, USA). 15, 30 and 60 minutes after injection, liver and gastrocnemius muscle were collected and snap frozen in liquid nitrogen for phospho-protein analysis. Phospho-IR was measured using an ELISA kit (Tyr 1150/1151; Cell Signaling Technologies, MA, USA) and a phospho-Akt (Ser 473; Meso Scale Discovery, MD, USA) assay kit according to manufacturer's instructions. Results at three time points are overall comparable and 30-minute measurements are shown in the Results section.

### Plasma and Tissue Cmpd1 Measurements

Animals were dosed i.p. at 30 mg/kg followed by bleeding and tissue extractions at 30, 60 and 240 min post dose. Plasma and tissues drug levels were determined by LC-MS/MS following protein precipitation with acetonitrile as described before [25].

### Statistical Analysis

Data analyses were performed in GraphPad Prism (GraphPad Software, San Diego, CA). Calculations of p-values were based on analysis of variance (ANOVA) and the unpaired student's *t* test, whichever was applicable. Statistical significance was defined as p < 0.05.

## Results

### Cmpd1 Potentiates Insulin-mediated Glycemic Control

**Fig 1:**
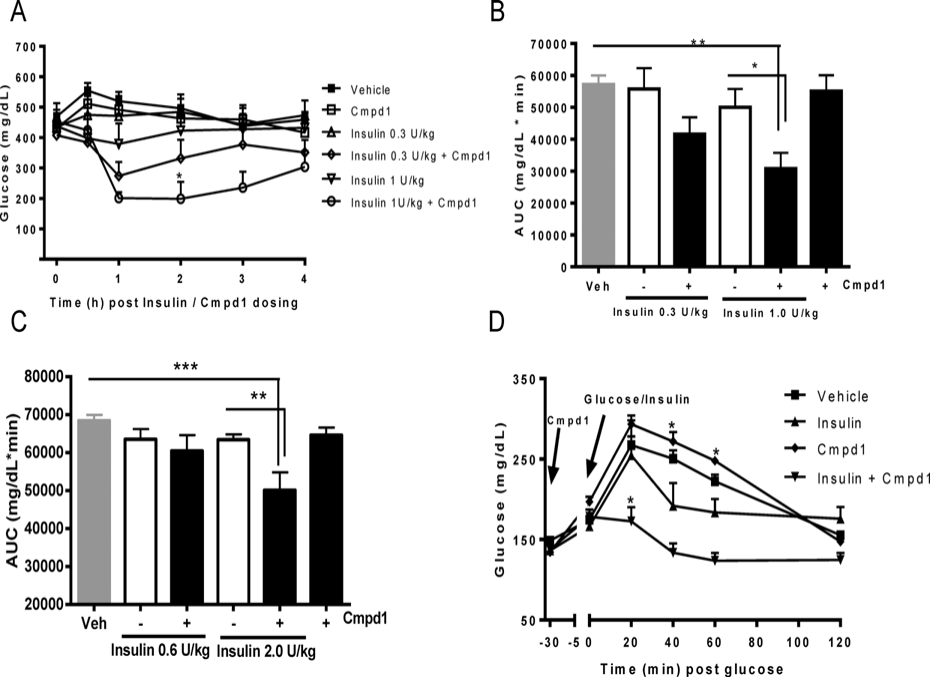
Cmpd1 potentiates insulin-mediated glycemic control. (A) Glucose lowering in STZ-diabetic mice. Cmpd1 (30 mg/kg) and insulin (0.3 or 1 U/kg) were dosed at time 0. Glucose was measured hourly for 4 hours. *p < 0.05 vs. insulin 1 U/kg. (B) Glucose AUC (0–4 h) calculated according to data in (A). *p < 0.05 and **p<0.01 vs. comparators as indicated. (C) Glucose lowering in db/db mice were conducted similarly as in (A). Cmpd1 (30 mg/kg) and insulin (0.6 or 2 U/kg) were dosed and AUC (0–4 h) calculated accordingly. **p<0.01 and ***p<0.001 vs. comparators as indicated. (D) Glucose tolerance test in normoglycemic mice. Cmpd1 (30 mg/kg) was dosed i.p.at—30 minutes and 5 g /kg glucose (p.o.) and insulin (0.5 U/kg; i.p.) were administered at time 0. Glucose was measured at specified time points up to 120 min. *p < 0.05 and **p<0.01 vs. insulin. The data are means ± SEM with n = 8 in each group.

The effects of Cmpd1 on glucose lowering were first tested in STZ-diabetic mice. When administered alone, Cmpd1 at doses up to 30 mg/kg did not lower glucose (Fig. 1A). Addition of insulin to Cmpd1 led to more potent glucose lowering than with corresponding doses of insulin alone, reaching 46% AUC reduction at 1 U/kg insulin when compared with insulin only group (Fig. 1A-B).

Identical studies conducted in hyperglycemic db/db mice produced similar results, even though the mice were more insulin resistant than STZ-diabetic mice. Cmpd1 potentiated insulin-mediated glucose lowering in db/db mice, showing a clear additive effect with insulin (Fig. 1C). The combination group displayed 27% lower glucose AUC compared to mice treated with 2 U/kg insulin alone.

The effects of Cmpd1 and insulin combination were also studied in normoglycemic C57BL/6 mice after injections of glucose and insulin. As shown in Fig. 1D, 30 mg/kg cmpd1 alone did not improve glucose tolerance. When combined with insulin, however, Cmpd1 significantly enhanced insulin-induced glucose disposal.

### Combination of Cmpd1 with Insulin Does Not Increase Hypoglycemia Risk

**Fig 2:**
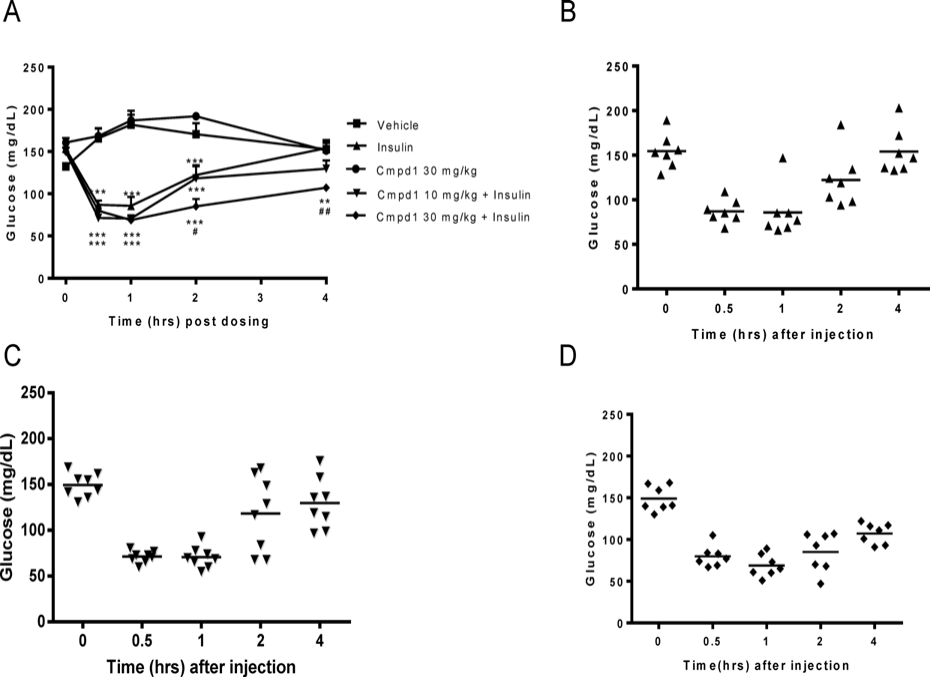
Combination of cmpd1 with insulin in normoglycemic mice extends glucose lowering duration of action but does not increase hypoglycemia risk. (A) Glucose lowering in normoglycemic C57BL/6 mice. Cmpd1 (10 or 30 mg/kg) and insulin (1 or 3 U/kg; only 3 U/kg data are shown) were dosed at time 0. Glucose was measured hourly for 4 hours. ** p<0.01 and *** p<0.001 vs. vehicle; # p<0.05 and ## p<0.01 vs. insulin alone. The data are means ± SEM with n = 8 in each group. Individual animal glucose values and their means of 3 U/kg insulin alone (B), 3 U/kg insulin plus 10 mg/kg Cmpd1 (C) or 3 U/kg insulin plus 30 mg/kg Cmpd1 (D) are shown. No significant increase of hypoglycemia was detected in the combination groups.

Simple increase of insulin potency alone is similar to dose elevation and could lead to a higher incidence of hypoglycemia, which is the major risk factor of marketed insulin and insulin analogs. The Cmpd1 and insulin combination studies in diabetic mice described above (Fig. 1A-C) clearly show potentiation of glucose lowering. Interestingly, there was no indication of higher hypoglycemia risk in combination-treated animals compared with corresponding insulin alone group. To evaluate the risk of hypoglycemia more carefully, Cmpd1 and insulin were administered in normoglycemic mice. Consistent with previous studies, Cmpd1 alone had no effect on glucose lowering, while a dose of insulin (1 or 3 U/kg) produced a robust glucose lowering with peak efficacy between 30 minutes and 1 hour post dosing (Fig. 2A; only 3 U/kg insulin results are shown). Unexpectedly, the addition of increasing doses of Cmpd1 to insulin (both 1 and 3 U/kg, which is a high dose for non-diabetic lean mice and at the edge of inducing hypoglycemia) had no effect on glucose trough, and did not produce any signs of hypoglycemia. This is true in the presence of the extended glucose lowering duration of action, as demonstrated by significantly lower glucose in Cmpd1 (at 30 mg/kg) plus insulin group 4 hours after dosing (Fig. 2).

Lack of hypoglycemia from the combination treatment can be better viewed in scattered plots (Fig. 2B-D) where the glucose values of individual animals are compared. From these, it appears that combining Cmpd1 (30 mg/kg) with insulin decreased the spread of glucose values among the individual animals rather than simply shifting the average glucose levels downwards. Plasma C-peptide (18888±1493 vs.24581+ 1251 pM*minute, p>0.05, n = 6) or glucagon (3187±268 vs. 3949±675 pg/mL*minute, p>0.05, n = 6) AUC between 0–60 min or at individual time points (data not shown) post dosing were not different between mice receiving insulin alone and the combination treatment.

### Cmpd1 Has Differential Effects on Insulin Signaling in Diabetic and Normoglycemic Tissues

**Fig 3:**
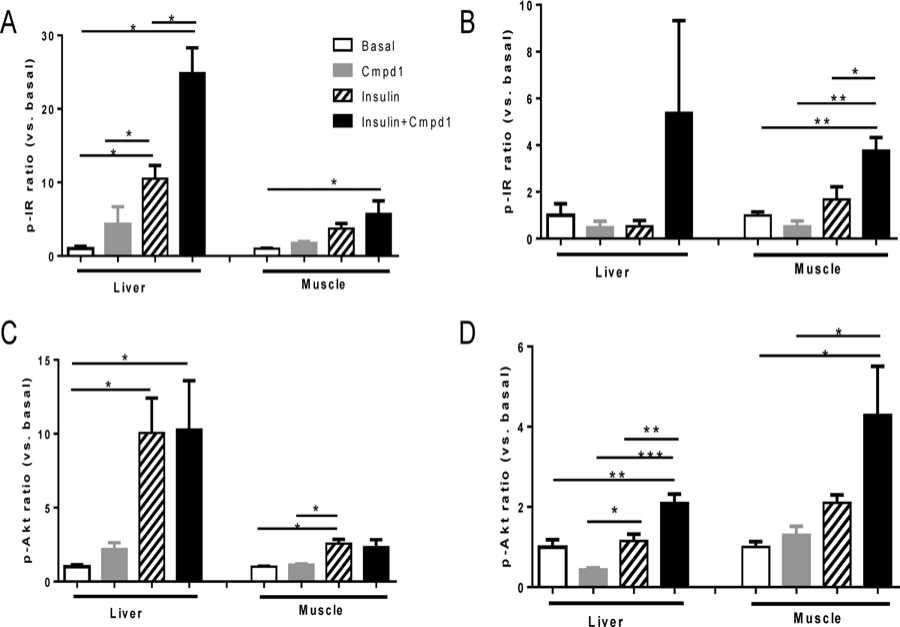
Cmpd1 has differential effects on insulin signaling in diabetic and normoglycemic tissues. Mice were fasted for 4 hours and received Cmpd1 i.p. and 1 U/kg insulin administration at time 0. 30 min post injection, liver and gastrocnemius muscle were collected to examine insulin signaling. Tissue lysate phospho-protein analysis was conducted using phosphor-IR (Y1150/1151) from Cell Signaling Technology and phospho-Akt (Ser473) Assay kit from Meso Scale Technology. Fold changes of compound treated vs. basal untreated samples were calculated for pIR normoglycemic mice (A), pIR diabetic mice (B), pAkt normoglycemic mice (C) and pAkt diabetic mice (D). Basal liver pIR and pAkt levels in STZ-diabetic mice were ~3 fold higher than that in normoglycemic mice but overall magnitude of stimulation by insulin or Cmpd1 insulin combination are comparable in the two models. Basal muscle pIR and pAkt levels are similar in the two models. Results shown are means ± SEM. *p < 0.05, **p < 0.01, ***p < 0.001 vs. comparators as indicated; n = 4 per condition.

To elucidate the mechanism of glucose lowering potency increase without additive hypoglycemia risk mediated by the combination of Cmpd1 and insulin, tissue specific IR and Akt phosphorylation levels were compared in normoglycemic and STZ-diabetic mice. In order to compare the effects across all conditions, fold changes of pIR and pAkt over basal condition were calculated. In normoglycemic C57BL/6 mice, insulin and its combination with Cmpd1 showed a larger increase in both pIR and pAkt in liver vs. skeletal muscle (Fig. 3A and 3C), suggesting liver was the major site of insulin action in healthy normoglycemic mice. In contrast, pIR and pAkt levels in STZ-diabetic mice indicated relatively reduced liver action (absolute fold increase in liver and relative to muscle; largely due to increased basal levels in the STZ-diabetic mice) by insulin alone (Fig. 3B and 3D). Combining Cmpd1 with insulin appears to have a larger impact on liver pIR and pAkt increase (vs. insulin alone) in STZ-diabetic mice than in normoglycemic mice (Fig. 3).

When insulin signaling is compared between normoglycemic and diabetic mice, it is clear that Cmpd1 enhances pIR in both lean and STZ mice (Fig. 3A & 3B). In contrast, the effect of the Cmpd1 plus insulin combination on pAkt is significantly different from its effect on IR phosphorylation. Cmpd1 increased insulin’s effects on pAkt in STZ-diabetic mice (Fig. 3D), but had totally blunted effects on pAkt in normoglycemic mice (Fig. 3C), in both liver and muscle. These results suggest that Cmpd1 affects downstream signaling of IR in a way that makes the animals responsive to circulating glucose levels at the level of Akt phosphorylation. In other words, Cmpd1 sensitizes insulin action in a glucose responsive manner rather than being simply additive to insulin’s glucose lowering efficacy.

### Glucose Lowering induced by Cmpd1 and Insulin Combination is IR Dependent

**Fig 4:**
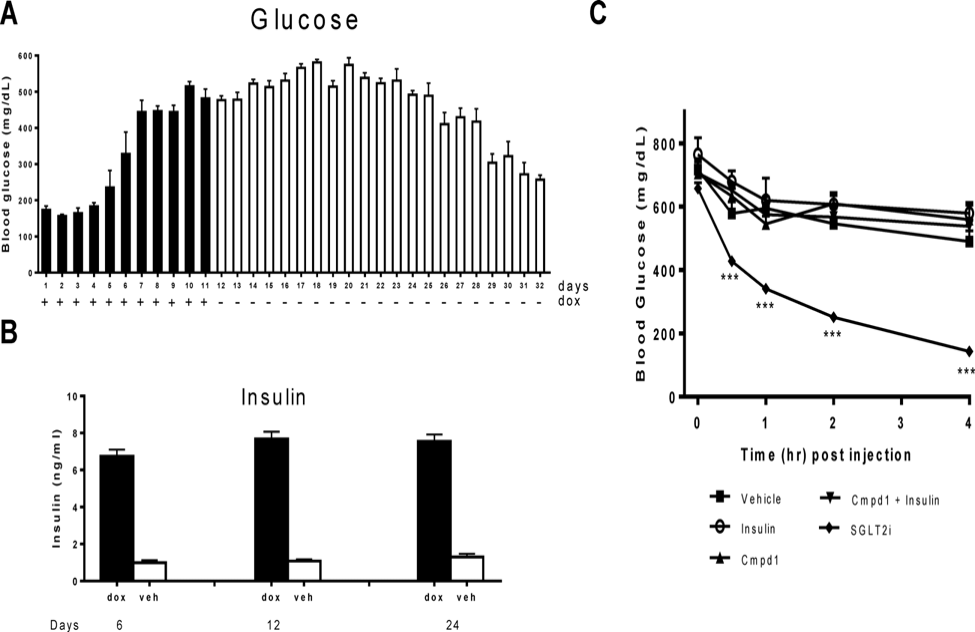
Glucose lowering by insulin or its combination with cmpd1 is IR dependent. Mice expressing IR-specific shRNA were fed with doxycycline in drinking water from day 1–11. Blood glucose (A) was monitored daily for 32 days during and post doxycycline treatment. Plasma insulin in doxycycline treated mice was examined on day 6, 12 and 24 in comparison with vehicle treated mice (B). n = 8 in each group. (C) Hyperglycemic IR-shRNA mice 15 days post doxycycline treatment received acute treatment of Cmpd1 (30 mg/kg) and insulin (1 U/kg) individually or in combination. A SGLT2 inhibitor (SGLT2i) was used as a control. Blood glucose was monitored hourly after dosing for 4 hours. ***p < 0.001 vs. vehicle-treated animals. The data are means ± SEM with n = 8 in each group.

Cmpd1 was identified from an affinity screen and has been shown to interact with IR *in vitro* [22]. To demonstrate that its glucose lowering effect is dependent on IR *in vivo*, Cmpd1 was studied in mice that have an inducible whole body knockdown (KD) of IR (iIR-KD) mediated by IR shRNA [24]. As shown in Fig. 4, and consistent with previous reports [24], global IR KD induced by doxycycline treatment (see Materials and Methods and Ref. 24 for more experimental details) led to hyperglycemia and hyperinsulinemia within seven days, which could be maintained for up to 2 weeks even after doxycycline withdrawal (Fig. 4A-B). Cmpd1 alone or its combined with insulin showed no significant glucose lowering in iIR-KD mice 15 days after doxycycline withdrawal, indicating that the *in vivo* insulin sensitizing efficacy of cmpd1 is IR-dependent (Fig. 4C). Even though severely hyperglycemic, iIR-KD mice could respond to glucose lowering mechanisms not dependent upon the insulin receptor. As an example, robust glucose lowering by a SGLT2 inhibitor was demonstrated in the same mice (Fig. 4C) [37].

### Cmpd1 is Enriched and Has Slow Clearance in Liver

Plasma and tissue levels of Cmpd1 were measured after i.p. injection of the compound alone (30 mg/kg) or together with 1 U/kg of insulin. Cmpd1’s clearance was faster in plasma than in liver or muscle (Table 1). Compared with muscle, liver accumulated significantly higher levels of Cmpd1 and it was metabolized slower than in muscle, as demonstrated by comparable liver levels of Cmpd1 at 30 and 240 minutes post dosing (Table 1). Because liver is one major site of action by Cmpd1 insulin combination, these data are consistent with the observed extension of insulin action by Cmpd1 in glucose lowering (e.g. Fig. 2A).

**Table 1:** Cmpd1 drug levels in mouse plasma and tissues.

		C57BL/6 Mice			STZ Diabetic Mice		
	Time (min)	Plasma	Liver	Muscle	Plasma	Liver	Muscle
Cmpd1	30	13.9±4.5	26.2±9.3	0.49±0.3	20.2±7.6	12.5±0.9	1.21±0.5
	60	4.6±4.2	19.8±8.0	0.52±0.3	9.1±7.8	9.0±3.1	1.19
	240	<0.09	13.9±2.0	0.61	0.60	9.9±0.8*	0.49
Cmpd1 + Insulin	30	27.7±13.9	14.6±2.6	0.53±0.3	12.4±10.5	11.3±1.4	2.09±1.3
	60	14.9±7.4	16.5±3.4	0.59±0.2	3.7±5.3	11.3±1.7	0.96±0.5*
	240	4.7±2.7	13.8±3.1	0.62±0.2	<0.09	9.3±1.7	0.67

Cmpd1 (30 mg/kg; in PBS) and insulin (1 U/kg) were dosed i.p. (n = 3)

Serial bleeds and tissue extractions at 30 min, 60 min and 240 min post dose

*: p < 0.05 vs. corresponding C57BL/6 mice.

## Discussion

The insulin receptor is one of the best validated targets for glycemic control. Recombinant insulin and its analogs represent one of the most important classes of therapeutics for diabetes, and have been prescribed to nearly one third of U.S. anti-diabetic drug users in the past decade [1,2]. However, unfavorable perceptions regarding frequent and lifestyle impacting injections and the potential adverse effects associated with insulin usage, such as hypoglycemia, have limited the wider adoption of insulin products, and have consequently hindered better glycemic control in patients [3]. The availability of an orally active insulin mimetic would be attractive to diabetics. That is why efforts to identify small molecule IR activators have never stopped, despite significant challenges [9–11,15–19]. The recent surge of activity to develop orally delivered insulin analogs [26] and additional alternative delivery approaches [27, 2] underscore the desire to activate IR using more convenient and less intrusive methodologies. However, most of these efforts have not addressed the hypoglycemia associated with IR activation in a fundamental way.

Positive allosteric modulators have been successfully developed for GPCRs [28,29], and some reports indicate the possibility of applying the same approach to receptor tyrosine kinases [28,30]. The recent identification of monoclonal antibodies that bind to IR and function as insulin sensitizers supports the feasibility of targeting IR for insulin sensitization [31]. Allosteric modulators of IR that function depending on the presence of insulin would allow patients to use their own insulin more effectively (e.g. in type 2 diabetics), or to inject lower insulin doses (e.g. in type 1 patients), leading to fewer adverse events such as hypoglycemia and body weight gain. Data from our studies with Cmpd1 provide evidence that it behaves like an insulin sensitizer *in vivo*, i.e. neutral on glucose by itself but strongly enhanced insulin potency on glycemic control (e.g. glucose lowering, improvement of insulin sensitivity and glucose tolerance) when the two agents are administered to the same animals. In addition, this Cmpd1-mediated synergistic effect on glucose lowering diminishes unexpectedly when glucose approaches hypoglycemia levels, even though Cmpd1 extended insulin’s duration of action significantly under the same conditions (Fig. 2). These unique properties suggest Cmpd1 may be able to engage a mechanism that is responsive to glucose.

The insulin sensitizing and glucose sensing activity of Cmpd1 could be due to several potential mechanisms. As shown in Fig. 2, the C-peptide or glucagon levels of mice treated with a combination of Cmpd1 and insulin were not significantly different from those of mice receiving insulin alone, ruling out the possible involvement of feedback regulation of insulin secretion or glucagon-mediated counter-regulation. This is in line with expectations, since Cmpd1 did not further decrease glucose levels beyond what is observed with insulin alone in normoglycemic mice (Fig. 2). In contrast, it appears that the major impact of Cmpd1 in this setting is to extend insulin’s duration of action and to lower glucose variability (compare Fig. 2D vs. 2B). Pertaining to this, it is possible that Cmpd1’s specific tissue distribution could play a role. Levels of Cmpd1 in liver are significantly higher (and more stable over time) than in peripheral tissues, such as muscle (Table 1), which could result in an insulin sensitization effect that is more liver-centric. This is likely the case, as a larger fold increase in liver pIR was detected in the combination group, especially in non-diabetic mice (Fig. 3A). Liver preference has been proposed as a key mechanism that underlies the lower hypoglycemia risk associated with physiological insulin, which subcutaneously delivered insulins generally lack and contributes to their elevated hypoglycemia risk compared to endogenous insulin [32–34]. This known liver preference arises from the fact that insulin released from pancreas has higher exposure to liver compared to peripheral tissues (e.g. skeletal muscle) due to significant liver extraction and creation of an insulin gradient in portal vein vs. peripheral vein. The differential pIR activities associated with the insulin and Cmpd1 combination in liver vs. peripheral tissues (i.e. higher insulin tone in liver vs. muscle) mimics endogenous insulin tissue actions, potentially contributing to reduced hypoglycemia risk.

Another possible explanation for the apparent glucose sensing action of Cmpd1 could be related to its effects on IR downstream signaling, which arise from its binding to an IR intracellular region close to the kinase domain and multiple Tyr phosphorylation sites [7,35]. Phosphorylation of these Tyr sites are critical for diverse actions mediated by IR, such as downstream signaling [7] and endocytosis [35]. When compared to insulin alone, the combination of Cmpd1 and insulin produces minimal effects on pAkt (in contrast to changes in pIR) in normoglycemic mice (Fig. 3C vs. 3A), suggesting that Cmpd1’s synergistic effect on insulin action is lost when blood glucose levels reach euglycemia. While the underlying molecular mechanism is not obvious, further studies will provide better understanding of this interesting observation and guide identification of next generation leads. For example, Cmpd1 binding to IR may help to generate “biased” signaling by blocking certain downstream events (e.g. interaction with adaptor proteins, kinase or phosphatase complex), or by inducing an unusual IR conformational change upon binding of insulin to the receptor. It is also not clear whether the observed phenomenon is due to the action of Cmpd1 alone, or whether the involvement of additional factors is also required. Along these lines, glucose has been reported to bind to and modulate insulin-IR binding [36]. Even though this glucose effect may not be robust under normal conditions, it could be more readily detectable when IR is bound to an allosteric modulator and adopts a different conformation. Any potential involvement of off-target interactions of Cmpd1 in the current study could be ruled out, as its effect on glucose lowering is blunted in IR knockdown mice (Fig. 4).

In summary, the *in vivo* studies presented here suggest that it is possible to modulate insulin receptor phosphorylation and activity pharmacologically to increase insulin sensitivity, in a way that is distinct from conventional insulin sensitizing mechanisms. Therapeutics derived from IR allosteric binders could provide benefits of improved potency without increased hypoglycemia risk and ease of administration compared to current standard of care, and thus could provide distinct and useful additions to current treatment options.
